# A Rarely Seen Multilevel Thoracic Vertebral Fracture after a Nocturnal Hypoglycemic Convulsion Attack

**DOI:** 10.1155/2015/646352

**Published:** 2015-04-05

**Authors:** Ebru Atalar, Cuneyd Gunay, Hakan Atalar, Tugba Tunc

**Affiliations:** ^1^Department of Physical Theraphy and Rehabilitation, Ankara Ataturk Chest Diseases and Chest Surgery Training and Research Hospital, 06280 Ankara, Turkey; ^2^Department of Orthopaedics and Traumatology, Faculty of Medicine, Eskisehir Osmangazi University, 26480 Eskisehir, Turkey; ^3^Department of Orthopaedics and Traumatology, Gazi University School of Medicine, 06560 Ankara, Turkey; ^4^Department of Neurology, Gazi University School of Medicine, 06560 Ankara, Turkey

## Abstract

A 49-year-old male presented with acute midthoracic severe back pain following a witnessed nocturnal convulsion attack. There was no history of trauma and the patient had a 23-year history of Type I diabetes mellitus. MRI scans of the thoracic spine revealed compression fractures at T5, T6, T7, and T8 vertebrae. The patient was treated conservatively. At 17 months after the initial diagnosis, the complaints of back pain had been resolved and the patient was able to easily undertake daily living activities. Hypoglycaemia is a common problem in diabetic patients treated with insulin. Convulsions may occur as a consequence of insulin-induced hypoglycemia. Nontraumatic compression fractures of the thoracic spine following seizures are a rare injury. Contractions of strong paraspinal muscles can lead to compression fracture of the midthoracic spine. Unrecognized hypoglycaemia should be considered to be a possible cause of convulsions in insulin-dependent diabetic patients. The aim of this report is to point out a case of rarely seen multilevel consecutive vertebrae fractures in a diabetic patient after a nocturnal hypoglycaemic convulsion attack.

## 1. Introduction

Seizures increase the risk of fracture throughout the skeleton by muscular contractions even without any direct trauma [1, 2]. Fractures and dislocations caused by a seizure have also been described, dislocations of the hip with or without fractures of the acetabulum or the femoral head or femoral neck, compression or burst fractures of the vertebrae, dislocations with or without fractures of the temporomandibular joint, and also dislocations of the shoulder joint with or without fractures [3, 4]. It has been reported that fractures are 2–6 times more common in epileptic patients relative to the general population [4, 5].

Nontraumatic fractures mostly present a diagnostic dilemma. Fracture risk is less reported in nonepileptic seizures. Various metabolic conditions leading to a decrease in bone mineral density may also cause fractures [6]. Hypoglycemia is a common side-effect of insulin therapy and is associated with significant mortality and morbidity rates in the diabetic population [7]. However, musculoskeletal injuries related to hypoglycaemic convulsions have seldom been reported, including vertebral fractures, joint dislocations, or bone fractures [7, 8].

Vertebral fractures caused solely by a convulsive seizure have rarely been reported in literature [2]. The patient in this report is described as having multilevel vertebral fracture following a seizure without any history of epilepsy.

## 2. Case Report

A 49-year-old retired male was admitted to the physical therapy outpatient clinic with severe back pain. From the history, it was learned that he had an acute back pain in the morning with no trauma. On the day before presentation, he had suffered a witnessed nocturnal attack, of which he had no recollection. The patient was oriented normally on arrival and reported that he had not experienced any previous nocturnal attacks like this, which was confirmed by his wife. The attack was described by his wife as follows: he started to lock his jaw, stiffened, was sweating, convulsed, and became unconscious. The duration of the seizure was about 3 minutes. Following the seizure he was briefly postictal, during which time his wife noted decreased movement of his arms and lower extremities. She reported that after the seizure he slept again and, in the morning, he had a back pain. There was no history of trauma, previous falls, prior seizures, stroke, syncope, or steroid medication. In the absence of a history of trauma, an epileptic seizure was suspected. It was learned that he had a 23-year history of Type I diabetes mellitus. For the last 20 years, he had required Humulin R three times daily (11 IU/at morning, noon, and evening), and Lantus once a day (22 IU/at midnight). Further medical history was not relevant and the patient had no history of epilepsy. The patient was not using antiepileptic drugs (AED) or corticosteroids at that time. The cardiovascular and neurological examination results were normal. There were signs of a bitten tongue. The patient had complaints of dorsal vertebrae pain, which was determined by palpation on the dorsal vertebrae during physical examination.

**Figure 1:**
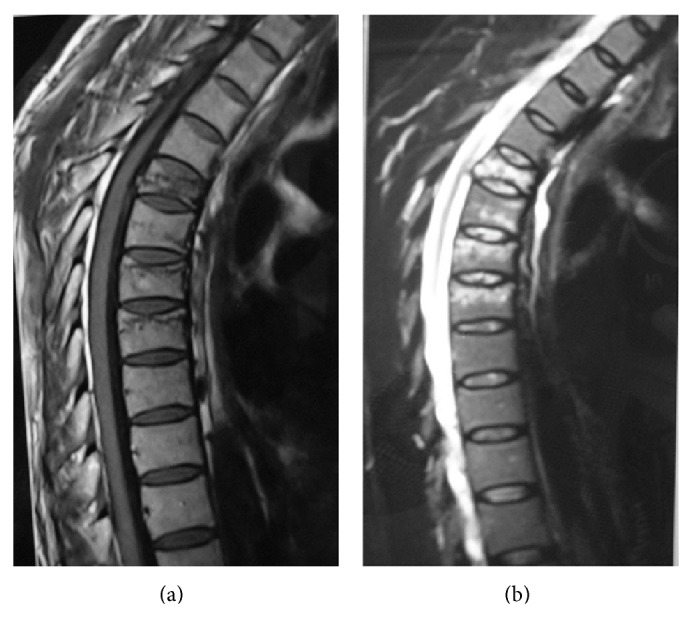
T1-weighted and T2-weighted sagittal magnetic resonance imaging (MRI) of the thoracic spine showing compression fracture at T5, T6, T7, and T8.

Vital signs were noted as follows: blood pressure: 125/80 mm/Hg, heart rate: 75/min., temperature: 36.5°C, respiratory rate: 18/min., weight; 70 kg., and height: 1.78 cm. Basic blood examination was normal except for severely raised fasting blood glucose: 216 mg/dL (65–107 mg/dL), slightly raised urea: 54 mg/dL (15–44 mg/dL), ALP: 146 U/L (30–120 U/L), and hemoglobin A1c: 6.7% (4.0–6.5%). Thoracic magnetic resonance imaging (MRI) was applied to evaluate the severity of the injury. Acute phase compression fractures on T5 (loss of 50% of vertebral height) and on T6, T7, and T8 vertebrae were determined and an additional slightly increased risk of thoracic kyphosis was reported (Figure 1).

**Figure 2:**
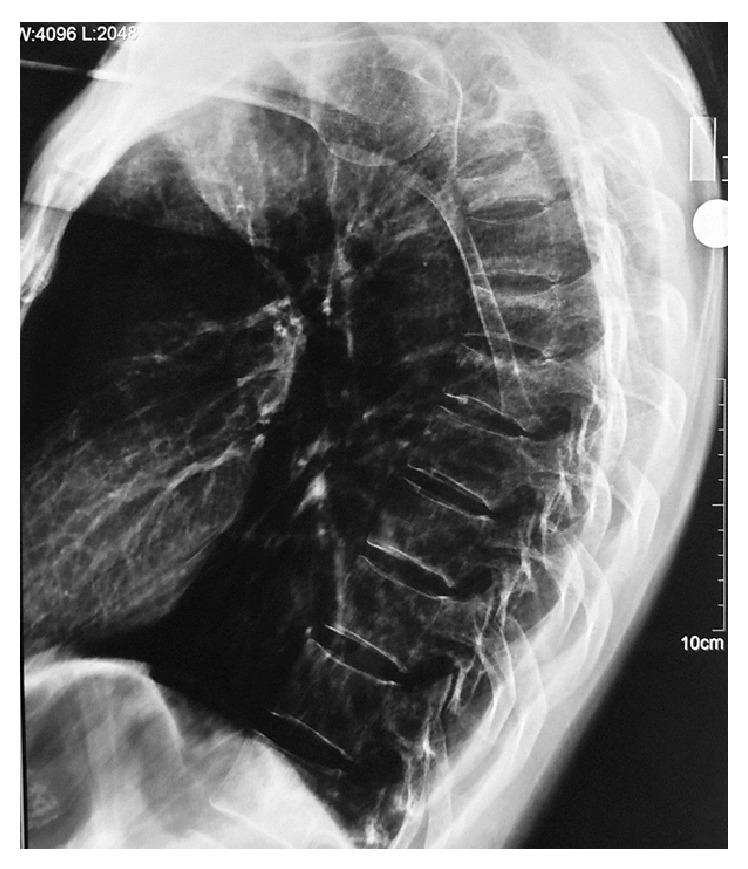
Lateral radiograph of thoracic vertebrae 4 months after the nocturnal convulsion attack.

For further evaluation, the patient was referred to the Neurology Department. In addition to basic studies, detailed investigations showed normal levels of parathyroid hormone, 25-hydroxy-vitamin D3, thyroid-stimulating hormone, free T3, free T4, cortisol, vitamin B12, and folate levels. Serum levels of calcium and phosphate were also within normal limits. Investigations for epilepsy were performed. An electroencephalogram (EEG) and a cerebral MRI showed no abnormalities. The patient was referred to the Orthopaedic Department, where he was treated conservatively. At the first follow-up examination, Dual Energy X-ray Absorptiometry (DEXA) was applied to rule out major osteoporosis due to diabetes. The T scores were as follows: femoral neck: −1.5, trochanter: −1.1, total: −1.2, lumbar (L) 1: −3.1, L2: −3.3, L3: −3.5, L4: −3.7, and total: −3.4. The DEXA reported osteopenia and osteoporosis on the right hip and in the lumbar spine, respectively. The osteopenia in the femoral region and osteoporosis in the lumbar region were treated with a single dose zoledronic acid 4 mg/5 mL intravenous infusion (for 1-year treatment), calcitonin 200 IU 1 × 1, and active vitamin D 0.25 mcg 1 × 1. Bed rest was applied for 3 weeks after the initial diagnosis, and then a thoracolumbar orthosis was used for 4 months (Figure 2). The patient started taking bisphosphonate at the time of diagnosis, and still continues to date. After 2 months, the patient was able to walk confidently with assistance. He made a rapid recovery and minimal deformity and little disability remained. At the most recent follow-up (17 months from the initial diagnosis), he had no complaints of back pain. Written informed consent was obtained from the patient for publication of this case report and the accompanying images.

## 3. Discussion

Reviews in literature have identified a number of etiologies causing multilevel fractures of the vertebrae, with trauma being the leading cause but consecutive multilevel nontraumatic fractures, as in the present case, have been reported only once. To the best of our knowledge, this is the second report of multilevel fractures of vertebrae after a hypoglycemic nocturnal convulsion attack in English literature. Nabarro [8] reported 4 cases of vertebrae fractures following hypoglycemic seizures, one of which was similar to the current case.

Fracture risk is notably increased in epileptic patients, with the risk having been shown to be as high as 43% [9]. Seizures can induce fractures of the femoral neck, humeral head, acetabulum, scapula, or vertebral column by violent contractions of the skeletal muscles [10]. In addition, the use of antiepileptic drugs (AED) in epileptic patients increases the risk of fracture [11]. Nocturnal hypoglycaemia is common but often unrecognized in insulin-dependent diabetic patients [12, 13]. It has been reported that 10% of the diabetic population have sustained a musculoskeletal or head injury due to hypoglycemia, and 7.9% have had one or more hypoglycaemic convulsions [14]. Despite this high prevalence, the relatively low rates of fractures after diabetic convulsions reported in literature is possibly due to no association of a relationship between diabetes mellitus and musculoskeletal injuries. Most vertebral fractures caused by a seizure are inherently stable with no neurological deficit. When a vertebral fracture is symptomatic the patient usually complains of back pain.

Severe hypoglycemia is an obvious danger for diabetic patients. Hart et al. reported that causes of hypoglycemia in diabetic patients include alcohol consumption, strenuous exercise, too much insulin, inadequate dietary intake of carbohydrate, and other unidentifiable reasons [14]. The above-mentioned known causes were discounted in the current case, leaving unidentified reasons. When hypoglycaemia occurs during sleep the patient is often unaware of the autonomic warning symptoms, and convulsions induced by hypoglycaemia may be mistakenly diagnosed as idiopathic epilepsy. However, in the current case, epilepsy was initially suspected, and the patient was referred to neurology department for tests.

The avoidance of subclinical hypoglycaemia during the night may require the occasional random measurement of capillary blood glucose by diabetic patients as part of their routine of blood glucose monitoring at home. This may awaken both the patient and the doctor to the reality of nocturnal hypoglycaemia and its complications. The purpose of this case report was also to emphasize the importance of the emergency physician evaluating the seizure patient with a wide differential diagnosis, including a diabetes-related hypoglycaemic attack.

Bone mineral density in the lumbar spine and the femoral neck was seen to have decreased, possibly due to Type I diabetes. Some prospective data have shown that diabetes mellitus is a statistical risk factor for osteoporosis, especially in cases of insulin-treated diabetes, a long history of diabetes, or in the elderly [5]. The present case demonstrates that forceful muscle contractions during convulsive seizures can result in vertebral fractures without any external trauma. Muscle contractions during a seizure have been reported to result in vertebral fractures, especially at the thoracic levels [2]. Fractures resulting from convulsions usually affect one or two of the third to eighth dorsal vertebrae, are usually associated with attacks during sleep, and are more common in men. This unique dispersion occurs because compressive forces during contraction of the muscles are concentrated along the anterior and middle columns of the midthoracic kyphotic curve [10]. Roberg studied the mechanics of these “flexion fractures” and why they occurred in the dorsal region. He suggested that they were associated with the strength of the spinal extensors in the dorsal region being less than in the cervical and lumbar spine and with the strength and mechanical advantage of the flexors working from the pelvis to the rib cage [8]. The limited mobility of the dorsal spine explains the rapid recovery and limited disability of this case.

Compression fractures of spinal dorsal vertebrae after hypoglycemia-induced convulsions have been described in diabetic patients [15, 16]. Although only one case of four adjacent level compression fractures of the thoracic vertebrae has been reported [8]. In addition, diabetic myopathy encompasses a spectrum of diseases, including muscle inflammation, ischemia, hemorrhage, infarction, necrosis, fibrosis, and fatty atrophy. It is usually seen with long-standing, poorly controlled diabetes [17]. However, in our patient the diabetes were being controlled with the current insulin treatment, and also he has no complaints about his muscles.

The possibility of diabetes should be kept in mind, in cases of multilevel vertebrae fractures after a nocturnal seizure with severe back pain. A detailed history, review of the vital signs, physical examination, and appropriate radiological investigations will help in making a true assessment of the causes. The absence of trauma and possible postictal consciousness confusion may prevent an early diagnosis. In conclusion, although a multilevel fracture of the consecutive thoracic vertebrae is rare, it is important not to underestimate the force of a seizure. A complaint of back pain after a convulsive seizure should prompt radiological investigation for vertebral fracture, even in the absence of external trauma.
